# Use of AMH in the Differential Diagnosis of Anovulatory Disorders Including PCOS

**DOI:** 10.3389/fendo.2020.616766

**Published:** 2021-02-03

**Authors:** Martina Capuzzo, Antonio La Marca

**Affiliations:** ^1^Department of Medical and Surgical Sciences for Mother, Child and Adult, University of Modena and Reggio Emilia, Modena, Italy; ^2^Clinica EUGIN, Modena, Italy

**Keywords:** anovulatory disorders, polycystic ovary syndrome, amenorrhea, anti-Müllerian hormone, premature ovarian insufficiency

## Abstract

Since the historical use of gonadotrophin and estradiol levels to define the different anovulatory disorders has shown some limitations, the use of other markers such as anti-müllerian hormone (AMH) has been proposed. This review addresses the role of AMH in the differential diagnosis of anovulatory disorders, especially focusing on its value in the prognostic characterization of their severity. Current limitations and future clinical applications are discussed.

## Introduction

Anovulatory disorders in women can be various. The main classification in use is adapted from the one proposed by the World Health Organization (WHO) and by the 1995 ESHRE Capri workshop group (1). The possible causes of anovulation are here categorized into three groups on the basis of serum gonadotrophin and estradiol levels. Besides having diagnostic purposes, such classification also aims at guiding the therapeutic approach, since each anovulation subtends different long term health consequences and ovulation restoring strategies. WHO 1 anovulatory dysfunction, which accounts for 5–10% of all anovulatory disorders, is characterized by low gonadotrophin and low estradiol serum levels (2). The underlining cause of this dysfunction is usually a hypothalamic suppression, which occurs in association with weight loss and negative energy balance, such as in patients suffering from anorexia nervosa and endurance athletes. WHO 2 anovulatory dysfunction, which accounts for the 80% of all the ovulatory disorders, presents normal gonadotrophin and estradiol levels. Polycystic ovary syndrome (PCOS), which is diagnosed on the basis of the Rotterdam consensus criteria (at least two between oligo-anovulation, clinical or biochemical hyperandrogenism and polycystic ovarian morphology, PCOM), represents its most frequent example (3). At last, WHO 3 anovulatory dysfunction is characterized by an ovarian reserve depletion with high gonadotrophin and low estradiol levels. If the patient is younger than 40 years old, this may indicate premature ovarian insufficiency (POI) (4).

Gonadotrophin and estradiol levels, however, are often overlapping in the various forms of anovulation, with no clear discriminatory thresholds established. Apart from their use, which can be limited by the above mentioned vagueness, several other markers have been proposed for the differential diagnosis of anovulatory dysfunctions. In particular, serum anti-Müllerian hormone (AMH) levels have been indicated as a potential tool in the discrimination of the various anovulatory disorders. In this article we aimed at reviewing the literature on AMH as a differential diagnostic marker in anovulatory diseases.

## AMH Function in the Ovary and at the Central Level

Anti-Müllerian hormone (AMH) is a dimeric glycoprotein belonging to the transforming growth factor-beta (TGF-β) superfamily (5). In men, AMH is secreted by the Sertoli cells of the testes, inducing the regression of Mullerian ducts (6, 7). In adult life, AMH is exclusively produced in the ovary by the granulosa cells surrounding the growing follicles, from early antral to small antral follicles phase (8–10). It is therefore thought that its serum levels are a reflection of a cohort of small growing follicles (11, 12), which reflects the number of residual primordial follicles, or the ovarian reserve (13).

AMH is a key-regulator of ovarian function. It is considered a local growth factor and acts in the cellular differentiation, since it has been demonstrated to have a paracrine inhibitory effect on the activation of folliculogenesis (5). *In vitro* and *in vivo* studies on mice were the first to show that in AMH knockout animals the transition from primordial into growing follicles with subsequent early depletion of the primordial follicle pool was enhanced (13). In humans, AMH causes a decrease in the follicle-stimulating hormone (FSH)-stimulated aromatase expression (14), and also reduces FSH receptor messenger RNA (mRNA) expression (15), with a consequent modulation in the ovarian follicular responses to gonadotrophins. Moreover, *in vitro* studies proved a modulation of the response to luteinizing hormone (LH) induced by AMH (5). A central action of AMH on GnRH neurons has also been hypothesized in mice observing the increased LH pulsatility in many cases of PCOS, in which circulating AMH levels are also often elevated (16).

## AMH in WHO 1 Ovulatory Dysfunction

In WHO 1 patients, either low, normal or slightly elevated AMH levels have been described.

Normal serum AMH levels have been reported in most of the studies on women with central secondary amenorrhea (17–20). La Marca et al. showed that in women with functional hypothalamic amenorrhea, AMH serum levels were similar to those found in normal controls. Moreover, there were no significant differences between the two groups in the number of 2–6 mm follicles, suggesting that initial follicle recruitment is not abolished in hypogonadotropic hypogonadism, with a stagnation of small antral follicles (17). No statistically significant difference of AMH serum levels between women with hypothalamic amenorrhea and anorexia nervosa and control group was observed in another case-control study conducted by Luisi et al. (18). Levels of AMH were within the normal range for age in a further study of van Elburg et al. on patients suffering from anorexia nervosa, confirming unaffected gonadotropin-independent growth of small preantral and early antral follicles in these patients. Under the same conditions of initial body weight, premorbid weight, duration of amenorrhea, duration of study participation and amount of prescribed medications, in patients suffering from anorexia nervosa the higher the AMH levels, the higher the probability of ovarian function recovery, indicating a possible prognostic role for AMH (21). The same evidence was observed in a group of seven patients with hypopituitarism during the years of adolescence, whose AMH serum concentrations were in the age-specific reference range, while in three out of four patients diagnosed in the infancy AMH serum concentrations were on or below the age-specific 25th percentile, with worse prognostic implications (22). On the contrary, in patients with long term profound gonadotrophin deficiency such as isolated hypogonadotropic hypogonadism and Kallmann syndrome, AMH levels were significantly lower when comparing affected patients to healthy controls. The subgroup of patients with the lowest FSH levels showed also the lowest AMH levels, showing the correlation between AMH levels and the severity of gonadotropin deficiency (23).

In a recent study by Alemyar et al., median AMH values were significatively higher in a population of patients with hypothalamic hypogonadism compared to healthy controls, but lower than AMH levels in the PCOS population. We hypothesize that the increase in AMH levels in the group of patients with hypothalamic hypogonadism might be due to the presence of a relatively large pool of antral follicles smaller than 2 mm in diameter, which are not counted during transvaginal ultrasonography although they secrete AMH (24).

## AMH in WHO 2 Ovulatory Dysfunction

Polycystic ovary syndrome (PCOS) represents the most frequent clinical manifestation of WHO 2 anovulatory dysfunction. Serum AMH is consistently higher in PCOS women (17, 25–30). For such reason, since serum AMH levels reflect the excess of small follicles which ultrasonography cannot detect, AMH has been proposed as a better marker in the diagnosis of PCOS than antral follicle count (AFC) (31–34). In a meta-analysis conducted by Iliodromiti et al., the specificity and sensitivity in diagnosing PCOS in the symptomatic women were of 79.4 and 82.8%, respectively, for a cutoff value of AMH of 4.7 ng/mL (31). Dewailly et al. separated asymptomatic women with PCOM to those with normal ovarian morphology in order to better calibrate the cutoff for the AMH value to distinguish patients with PCOS from normal women. A higher specificity (97 vs. 92%) and a better sensitivity (92 vs 81%) were demonstrated for a cutoff value of AMH of 4.9 ng/mL compared to AFC (32). Nevertheless, a universal diagnostic threshold for serum AMH in the diagnosis of PCOS has not yet been reached, and its use as an alternative for detecting PCOM in the diagnosis of PCOS has not been recommended by the new European Society of Human Reproduction and Embryology 2018 guidelines (35).

The reasons behind the elevated AMH levels of these categories of patients are different. In PCOS women there is a stagnation of AMH-producing follicles, with a stockpiling of transitional and classic primary follicles whose differentiation in the subsequent development phases is disrupted (36). Besides the elevated number of AMH-producing follicles, in these patients an increased production of AMH per single follicle has also been observed, with the mean level of AMH four times higher in granulosa cells from ovulatory PCOS and 75 times higher in granulosa cells from anovulatory PCOS patients in a study by Pellatt et al., demonstrating a correlation between AMH values and the severity of the syndrome with the possibility of ovulation restoration (37). Besides being a marker for the diagnosis of PCOS, AMH has also been thought to have a role in the pathogenesis of the disease. Both Visser and Homburg stated that the high AMH concentrations present in women with PCOS could play an integral role in causing anovulation due to AMH’s inhibitory influence on the actions of FSH that normally promotes follicular development from the small antral stage to ovulation (38, 39). According to Cimino et al., AMH increases GnRH-dependent LH pulsatility and secretion, with the consequent dysregulation of follicle growth (16). Moreover, AMH seems to correlate with the severity of the syndrome. Higher levels of AMH have been shown in amenorrhoeic than in oligomenorrhoeic women with PCOS, reflecting a more evident impairment in follicular development and granulosa cell function in the ovaries of amenorrhoeic than in those of oligomenorrhoeic PCOS women (17). According to Tal et al., increased AMH levels correlate more with PCOS severity in women with ultrahigh AMH (> 10 ng/mL) having greater prevalence of polycystic ovarian morphology, oligomenorrhea and amenorrhea than in women with AMH 5–10 ng/mL (40). Nevertheless, AMH has also been proposed as a marker for treatment monitoring in PCOS women. PCOS obese and overweight patients who showed improvements in reproductive function after weight loss had lower baseline AMH levels compared with those who did not respond (41). In a study evaluating response to treatment, the group who responded less well to induction of ovulation was the one with higher AMH levels (42).

## AMH in WHO 3 Ovulatory Dysfunction

WHO 3 anovulatory dysfunction is characterized by low or undetectable AMH levels (17, 43, 44). Being connected to the loss of ovarian function, this condition is also known as primary ovarian insufficiency (POI), premature ovarian failure (POF) or premature menopause (44) when this condition occurs before age 40. In such dysfunction, the follicle pool is depleted, with a consequent ovarian insufficiency whose cause can be various: genetic, autoimmune, and iatrogenic (2). With respect to other markers such as AFC, FSH, inhibin B and estradiol, AMH seems to better reflect the continuous decline of the oocyte/follicle pool with age (15). AMH levels showed to be significantly different between incipient ovarian failure (IOF), with regular menstrual cycles and elevated FSH, and transitional ovarian failure patients (TOF), with oligomenorrhea and elevated FSH, permitting the identification of the clinical degree of follicle pool depletion (45). AMH values are also reported to perform as a predictor of follicle presence in ovarian biopsies performed on patients with a premature ovarian failure (46). Moreover, in patients diagnosed with steroidogenic cell autoimmunity (SCA-POI), since the depletion of follicles begins from the antral follicle stage, a preserved ovarian follicle pool producing AMH can be found for several years after an ovarian insufficiency diagnosis (47). The maintenance of AMH levels in women with autoimmune POI is important since it has been observed that in the initial stages of the disease, characterized by the persistence of antral follicles despite amenorrhea and high serum gonadotropin levels, strategies of fertility preservation such as *in vitro* maturation (IVM) can be performed before the inevitable follicular depletion (48).

## Discussion

The Anti-Müllerian hormone (AMH), given its relationship with the follicular ovarian pool, is a reliable marker of ovarian reserve and its clinical use has recently been extended and emphasized. In particular, it has been proposed by different authors as a potential marker in the differential diagnosis of the various forms of anovulatory dysfunctions: it is usually normal in patients with hypogonadotropic anovulation (even if it can be also low or slightly elevated), high in normogonadotropic anovulations and low in hypergonadotropic anovulations (2, 7, 15). Due to its significantly elevated values in PCOS it has also been proposed as a marker for the diagnosis (31–34), even if a universal diagnostic threshold for serum AMH in the diagnosis of PCOS has not yet been reached (35).

Its use as a single marker in differentiating the different anovulatory forms, however, has been discussed. Since its levels can be increased in patients with hypothalamic hypogonadism whilst there is no increase in AFC, Alemyar et al. warned to avoid its use as a single biomarker for the characterization of an anovulatory disorder, highlighting the importance of measuring gonadotrophins and estradiol in order to avoid the misdiagnosis with WHO 2 anovulations (24). Nevertheless, AMH has proven to be insufficient in characterizing alone some anovulations such as WHO 1 anovulation disorder, since in such anovulation its values can vary widely. However, the underlying causes (congenital, functional or iatrogenic) of WHO 1 anovulation are not always specified in the literature, which can represent an important limitation. The evaluation of the differences in serum AMH between long-term vs. short-term etiologies could in the future be helpful in guiding the evaluation of AMH in WHO 1 anovulations.

Several evidences from the literature show that, rather than a diagnostic biomarker, AMH could have an important role as an index of severity and a guide for treatment of the various anovulatory disorders. A correlation between AMH values and the probability of ovulation restoration has been described, even if, to date, no predictive values in terms of ovulation and pregnancy for any ovulation-inducing treatment have been demonstrated for this assay.

An important limitation in the use of AMH as a single diagnostic marker in the differential diagnosis of anovulations is represented by different numerical calibration of the existing AMH assays. An international standard is needed to standardize the existing assays before diagnostic cutoffs are meaningful. The optimal performance and stability of the automated AMH assays now in use compared to previous manual assays is well recognized (49, 50), with many clinicians assuming that the values derived from the two most common automated assays, the Elecsys AMH assay and the Access AMH assay, are interchangeable (51). Nevertheless, a certain debate is still present in literature (51, 52). Another issue is the change of serum AMH level with age in the normal population, and hence an age-standardized value may be more appropriate in serving the diagnostic role. Defined study and control populations, biologically relevant cutoff values that reflect clustering of clinical features and are relevant to health outcomes and age-specific and improved accuracy and standardization of AMH assays are necessary before introducing AMH values as a diagnostic marker for PCOS (53).

In conclusion, AMH has been reported as a relevant diagnostic marker for anovulatory disorders. A debate in literature has been conducted over its candidability as a diagnostic marker in WHO 2 anovulations. However, at the moment its use for such purpose has not been recommended, while its employment in the characterization of the prognosis of the anovulations could be an important field of research and clinical application. Improvements in standardization of AMH assays and establishment of cutoff values based on large-scale validation in populations of different ethnicities and ages are needed.

## Author Contributions

MC did the bibliographic research and wrote the article. ALM revised the manuscript. All authors agree to be accountable for the content of the work. All authors contributed to the article and approved the submitted version.

## Conflict of Interest

The authors declare that the research was conducted in the absence of any commercial or financial relationships that could be construed as a potential conflict of interest.

## References

[1] Anovulatory infertility. The ESHRE Capri Workshop Group. Hum Reprod (1995) 10(6):1549–53. 7593533

[2] Lie Fong S Schipper I Valkenburg O de Jong FH Visser JA Laven JS The role of anti-Müllerian hormone in the classification of anovulatory infertility. Eur J Obstet Gynecol Reprod Biol (2015) 186:75–9. 10.1016/j.ejogrb.2015.01.007 25666342

[3] Rotterdam ESHRE/ASRM-Sponsored PCOS consensus workshop group Revised 2003 consensus on diagnostic criteria and long-term health risks related to polycystic ovary syndrome (PCOS). Hum Reprod (2004) 19(1):41–7. 10.1093/humrep/deh098 14688154

[4] European Society for Human Reproduction and Embryology (ESHRE) Guideline Group on POI Webber L Davies M Anderson R Bartlett J Braat D ESHRE Guideline: management of women with premature ovarian insufficiency. Hum Reprod (2016) 31(5):926–37. 10.1093/humrep/dew027 27008889

[5] Sacchi S D’Ippolito G Sena P Marsella T Tagliasacchi D Maggi E The anti-Müllerian hormone (AMH) acts as a gatekeeper of ovarian steroidogenesis inhibiting the granulosa cell response to both FSH and LH. J Assist Reprod Genet (2016) 33(1):95–100. 10.1007/s10815-015-0615-y 26631403 PMC4717146

[6] Moses MM Behringer RR A gene regulatory network for Müllerian duct regression. Environ Epigenet (2019) 5(3):dvz017. 10.1093/eep/dvz017 31579527 PMC6760261

[7] Oh SR Choe SY Cho YJ Clinical application of serum anti-Müllerian hormone in women. Clin Exp Reprod Med (2019) 46(2):50–9. 10.5653/cerm.2019.46.2.50 PMC6572668 31181872

[8] Wu CH Chen YC Wu HH Yang JG Chang YJ Tsai HD Serum anti-Müllerian hormone predicts ovarian response and cycle outcome in IVF patients. J Assist Reprod Genet (2009) 26(7):383–9. 10.1007/s10815-009-9332-8 PMC2758947 19768530

[9] Durlinger AL Visser JA Themmen AP Regulation of ovarian function: the role of anti-Müllerian hormone. Reproduction (2002) 124(5):601–9. 10.1530/rep.0.1240601 12416998

[10] Weenen C Laven JS Von Bergh AR Cranfield M Groome NP Visser JA Anti-Müllerian hormone expression pattern in the human ovary: potential implications for initial and cyclic follicle recruitment. Mol Hum Reprod (2004) 10(2):77–83. 10.1093/molehr/gah015 14742691

[11] de Vet A Laven JS de Jong FH Themmen AP Fauser BC Antimüllerian hormone serum levels: a putative marker for ovarian aging. Fertil Steril (2002) 77(2):357–62. 10.1016/s0015-0282(01)02993-4 11821097

[12] Fanchin R Schonäuer LM Righini C Guibourdenche J Frydman R Taieb J Serum anti-Müllerian hormone is more strongly related to ovarian follicular status than serum inhibin B, estradiol, FSH and LH on day 3. Hum Reprod (2003) 18(2):323–7. 10.1093/humrep/deg042 12571168

[13] Durlinger AL Gruijters MJ Kramer P Karels B Kumar TR Matzuk MM Anti-Müllerian hormone attenuates the effects of FSH on follicle development in the mouse ovary. Endocrinology (2001) 142(11):4891–9. 10.1210/endo.142.11.8486 11606457

[14] Chang HM Klausen C Leung PC Antimüllerian hormone inhibits follicle-stimulating hormone-induced adenylyl cyclase activation, aromatase expression, and estradiol production in human granulosa-lutein cells. Fertil Steril (2013) 100(2):585–92.e1. 10.1016/j.fertnstert.2013.04.019 23663993

[15] Pellatt L Rice S Dilaver N Heshri A Galea R Brincat M Anti-Müllerian hormone reduces follicle sensitivity to follicle-stimulating hormone in human granulosa cells. Fertil Steril (2011) 96(5):1246–51.e1. 10.1016/j.fertnstert.2011.08.015 21917251

[16] Cimino I Casoni F Liu X Messina A Parkash J Jamin SP Novel role for anti-Müllerian hormone in the regulation of GnRH neuron excitability and hormone secretion. Nat Commun (2016) 7:10055. 10.1038/ncomms10055 26753790 PMC4729924

[17] La Marca A Pati M Orvieto R Stabile G Carducci Artenisio A Volpe A Serum anti-müllerian hormone levels in women with secondary amenorrhea. Fertil Steril (2006) 85(5):1547–9. 10.1016/j.fertnstert.2005.10.057 16616745

[18] Luisi S Ciani V Podfigurna-Stopa A Lazzeri L De Pascalis F Meczekalski B Serum anti-Müllerian hormone, inhibin B, and total inhibin levels in women with hypothalamic amenorrhea and anorexia nervosa. Gynecol Endocrinol (2012) 28(1):34–8. 10.3109/09513590.2011.579664 21627557

[19] Jonard S Pigny P Jacquesson L Demerle-Roux C Robert Y Dewailly D The ovarian markers of the FSH insufficiency in functional hypothalamic amenorrhoea. Hum Reprod (2005) 20(1):101–7. 10.1093/humrep/deh560 15513979

[20] Li HW Anderson RA Yeung WS Ho PC Ng EH Evaluation of serum antimullerian hormone and inhibin B concentrations in the differential diagnosis of secondary oligoamenorrhea. Fertil Steril (2011) 96(3):774–9. 10.1016/j.fertnstert.2011.06.016 21737073

[21] van Elburg AA Eijkemans MJ Kas MJ Themmen AP de Jong FH van Engeland H Predictors of recovery of ovarian function during weight gain in anorexia nervosa. Fertil Steril (2007) 87(4):902–8. 10.1016/j.fertnstert.2006.11.004 17239876

[22] Sonntag B Nawroth F Ludwig M Bullmann C Anti-Müllerian hormone in women with hypopituitarism diagnosed before or during adolescence. Reprod BioMed Online (2012) 25(2):190–2. 10.1016/j.rbmo.2012.03.010 22683152

[23] Bry-Gauillard H Larrat-Ledoux F Levaillant JM Massin N Maione L Beau I Anti-Müllerian Hormone and Ovarian Morphology in Women With Isolated Hypogonadotropic Hypogonadism/Kallmann Syndrome: Effects of Recombinant Human FSH. J Clin Endocrinol Metab (2017) 102(4):1102–11. 10.1210/jc.2016-3799 28324034

[24] Alemyar A van der Kooi ALF Laven JSE Anti-Müllerian Hormone and Ovarian Morphology in Women With Hypothalamic Hypogonadism. J Clin Endocrinol Metab (2020) 105(5):dgaa116. 10.1210/clinem/dgaa116 32170295

[25] Cook CL Siow Y Brenner AG Fallat ME Relationship between serum müllerian-inhibiting substance and other reproductive hormones in untreated women with polycystic ovary syndrome and normal women. Fertil Steril (2002) 77(1):141–6. 10.1016/s0015-0282(01)02944-2 11779604

[26] Pigny P Merlen E Robert Y Cortet-Rudelli C Decanter C Jonard S Elevated serum level of anti-mullerian hormone in patients with polycystic ovary syndrome: relationship to the ovarian follicle excess and to the follicular arrest. J Clin Endocrinol Metab (2003) 88(12):5957–62. 10.1210/jc.2003-030727 14671196

[27] Laven JS Mulders AG Visser JA Themmen AP De Jong FH Fauser BC Anti-Müllerian hormone serum concentrations in normoovulatory and anovulatory women of reproductive age. J Clin Endocrinol Metab (2004) 89(1):318–23. 10.1210/jc.2003-030932 14715867

[28] Eldar-Geva T Margalioth EJ Gal M Ben-Chetrit A Algur N Zylber-Haran E Serum anti-Mullerian hormone levels during controlled ovarian hyperstimulation in women with polycystic ovaries with and without hyperandrogenism. Hum Reprod (2005) 20(7):1814–9. 10.1093/humrep/deh873 15802320

[29] Piltonen T Morin-Papunen L Koivunen R Perheentupa A Ruokonen A Tapanainen JS Serum anti-Müllerian hormone levels remain high until late reproductive age and decrease during metformin therapy in women with polycystic ovary syndrome. Hum Reprod (2005) 20(7):1820–6. 10.1093/humrep/deh850 15802325

[30] Wachs DS Coffler MS Malcom PJ Chang RJ Serum anti-mullerian hormone concentrations are not altered by acute administration of follicle stimulating hormone in polycystic ovary syndrome and normal women. J Clin Endocrinol Metab (2007) 92(5):1871–4. 10.1210/jc.2006-2425 17299061

[31] Iliodromiti S Kelsey TW Anderson RA Nelson SM Can anti-Mullerian hormone predict the diagnosis of polycystic ovary syndrome? A systematic review and meta-analysis of extracted data. J Clin Endocrinol Metab (2013) 98(8):3332–40. 10.1210/jc.2013-1393 23775353

[32] Dewailly D Gronier H Poncelet E Robin G Leroy M Pigny P Diagnosis of polycystic ovary syndrome (PCOS): revisiting the threshold values of follicle count on ultrasound and of the serum AMH level for the definition of polycystic ovaries. Hum Reprod (2011) 26(11):3123–9. 10.1093/humrep/der297 21926054

[33] Dewailly D Andersen CY Balen A Broekmans F Dilaver N Fanchin R The physiology and clinical utility of anti-Mullerian hormone in women. Hum Reprod Update (2014) 20(3):370–85. 10.1093/humupd/dmt062 24430863

[34] Lauritsen MP Bentzen JG Pinborg A Loft A Forman JL Thuesen LL The prevalence of polycystic ovary syndrome in a normal population according to the Rotterdam criteria versus revised criteria including anti-Mullerian hormone. Hum Reprod (2014) 29(4):791–801. 10.1093/humrep/det469 24435776

[35] Teede HJ Misso ML Costello MF Dokras A Laven J Moran L International PCOS Network. Recommendations from the international evidence-based guideline for the assessment and management of polycystic ovary syndrome. Fertil Steril (2018) 110(3):364–79. 10.1016/j.fertnstert.2018.05.004 PMC6939856 30033227

[36] Maciel GA Baracat EC Benda JA Markham SM Hensinger K Chang RJ Stockpiling of transitional and classic primary follicles in ovaries of women with polycystic ovary syndrome. J Clin Endocrinol Metab (2004) 89(11):5321–7. 10.1210/jc.2004-0643 15531477

[37] Pellatt L Hanna L Brincat M Galea R Brain H Whitehead S Granulosa cell production of anti-Müllerian hormone is increased in polycystic ovaries. J Clin Endocrinol Metab (2007) 92(1):240–5. 10.1210/jc.2006-1582 17062765

[38] Visser JA Themmen AP Anti-Müllerian hormone and folliculogenesis. Mol Cell Endocrinol (2005) 234(1-2):81–6. 10.1016/j.mce.2004.09.008 15836956

[39] Homburg R Crawford G The role of AMH in anovulation associated with PCOS: a hypothesis. Hum Reprod (2014) 29(6):1117–21. 10.1093/humrep/deu076 24770999

[40] Tal R Seifer DB Khanimov M Malter HE Grazi RV Leader B Characterization of women with elevated antimüllerian hormone levels (AMH): correlation of AMH with polycystic ovarian syndrome phenotypes and assisted reproductive technology outcomes. Am J Obstet Gynecol (2014) 211(1):59.e1–8. 10.1016/j.ajog.2014.02.026 24593938

[41] Thomson RL Buckley JD Moran LJ Noakes M Clifton PM Norman RJ The effect of weight loss on anti-Müllerian hormone levels in overweight and obese women with polycystic ovary syndrome and reproductive impairment. Hum Reprod (2009) 24(8):1976–81. 10.1093/humrep/dep101 19380385

[42] Pellatt L Rice S Mason HD Anti-Müllerian hormone and polycystic ovary syndrome: a mountain too high? Reproduction (2010) 139(5):825–33. 10.1530/REP-09-0415 20207725

[43] Faubion SS Kuhle CL Shuster LT Rocca WA Long-term health consequences of premature or early menopause and considerations for management. Climacteric (2015) 18(4):483–91. 10.3109/13697137.2015.1020484 PMC4581591 25845383

[44] Laven JS Primary Ovarian Insufficiency. Semin Reprod Med (2016) 34(4):230–4. 10.1055/s-0036-1585402 27513024

[45] Knauff EA Eijkemans MJ Lambalk CB ten Kate-Booij MJ Hoek A Beerendonk CC Dutch Premature Ovarian Failure Consortium. Anti-Mullerian hormone, inhibin B, and antral follicle count in young women with ovarian failure. J Clin Endocrinol Metab (2009) 94(3):786–92. 10.1210/jc.2008-1818 Epub 2008 Dec 9. Erratum in: J Clin Endocrinol Metab. 2010 Jan;95(1):465. 19066296

[46] Méduri G Massin N Guibourdenche J Bachelot A Fiori O Kuttenn F Serum anti-Müllerian hormone expression in women with premature ovarian failure. Hum Reprod (2007) 22(1):117–23. 10.1093/humrep/del346 16954410

[47] La Marca A Marzotti S Brozzetti A Stabile G Artenisio AC Bini V Italian Addison Network. Primary ovarian insufficiency due to steroidogenic cell autoimmunity is associated with a preserved pool of functioning follicles. J Clin Endocrinol Metab (2009) 94(10):3816–23. 10.1210/jc.2009-0817 19622621

[48] Grynberg M Jacquesson L Sifer C In vitro maturation of oocytes for preserving fertility in autoimmune premature ovarian insufficiency. Fertil Steril (2020) 114(4):848–53. 10.1016/j.fertnstert.2020.04.049 32709383

[49] Li HW Wong BP Ip WK Yeung WS Ho PC Ng EH Comparative evaluation of three new commercial immunoassays for anti-Müllerian hormone measurement. Hum Reprod (2016) 31(12):2796–802. 10.1093/humrep/dew248 27702835

[50] Pigny P Gorisse E Ghulam A Robin G Catteau-Jonard S Duhamel A Comparative assessment of five serum antimüllerian hormone assays for the diagnosis of polycystic ovary syndrome. Fertil Steril (2016) 105(4):1063–1069.e3. 10.1016/j.fertnstert.2015.12.023 26769302

[51] Tadros T Tarasconi B Nassar J Benhaim JL Taieb J Fanchin R New automated antimüllerian hormone assays are more reliable than the manual assay in patients with reduced antral follicle count. Fertil Steril (2016) 106(7):1800–6. 10.1016/j.fertnstert.2016.08.045 27692436

[52] Nelson SM Pastuszek E Kloss G Malinowska I Liss J Lukaszuk A Two new automated, compared with two enzyme-linked immunosorbent, antimüllerian hormone assays. Fertil Steril (2015) 104(4):1016–1021.e6. 10.1016/j.fertnstert.2015.06.024 26183313

[53] Teede H Misso M Tassone EC Dewailly D Ng EH Azziz R Anti-Müllerian Hormone in PCOS: A Review Informing International Guidelines. Trends Endocrinol Metab (2019) 30(7):467–78. 10.1016/j.tem.2019.04.006 31160167

